# Chitosan in viscosupplementation: in vivo effect on rabbit subchondral bone

**DOI:** 10.1186/s12891-017-1700-4

**Published:** 2017-08-15

**Authors:** R. Rieger, C. Boulocher, S. Kaderli, T. Hoc

**Affiliations:** 1 LTDS, UMR CNRS 5513, Université de Lyon, Ecole Centrale de Lyon, 36 av. Guy de Collongue, 69134 Ecully Cedex, France; 2 VetAgro Sup, University of Lyon, Veterinary Campus of VetAgro Sup, 69280, Marcy l’Etoile, France; 3 School of Pharmaceutical Sciences, University of Geneva and University of Lausanne, Quai Ernest-Ansermet 30, 1211 Geneva, Switzerland

**Keywords:** ACLT-induced osteoarthritis, Hyaluronic acid, Chitosan, Subchondral bone, Microarchitecture, Mineral density

## Abstract

**Background:**

To investigate the effect of intra-articular injection of Chitosan (Cs) added to hyaluronic acid (HA) on subchondral bone during osteoarthritis (OA), microarchitectural parameters and mineral density were measured in a rabbit model of early OA. A novel hybrid hydrogel adding reacetylated Cs of fungal origin to HA was compared to high molecular weight HA commercial formulation.

**Method:**

Eighteen rabbits underwent unilateral anterior cruciate ligament transection (ACLT) and were divided into three groups (Saline-group, HA-group and Hybrid-group) depending on the intra-articular injection compound. Eight contralateral knees were used as non-operated controls (Contralateral-group). Micro-computed tomography was performed six weeks post-ACLT to study subchondral bone microarchitectural parameters and mineral density at an early stage of OA development.

**Results:**

Cartilage thickness mean value was reduced only in Saline-group compared to Contralateral-group. When the Hybrid-group was compared to Saline-group, subchondral bone microarchitectural parameters (trabecular thickness and trabecular bone volume fraction) were significantly changed; subchondral bone plate and trabecular bone mineral densities (bone mineral density and tissue mineral density) were reduced. When the Hybrid-group was compared to HA-group, subchondral bone microarchitectural parameters (subchondral plate thickness and trabecular thickness) and trabecular bone mineral densities (bone mineral density and tissue mineral density) were significantly decreased.

**Conclusion:**

Conclusion: Compared to HA alone, the novel hybrid hydrogel, constituted of Cs added to HA, enhanced microarchitectural parameters and mineral density changes, leading to subchondral bone loss in a rabbit model of early experimental OA.

## Background

Osteoarthritis (OA) is a multi-causal disease that is difficult to mimic in complete in vitro and ex vivo models. Animal models remain most common for new therapeutic strategies development. Anterior Cruciate Ligament Transection (ACLT) in rabbits is a widely used experimental model to study OA pathogenesis and drugs efficacy [1–7]. Though no therapy to date has been approved as a disease modifying OA drug, several symptomatic slow acting OA drugs have demonstrated clinically relevant effects. One of the drugs falling into this category is viscosupplementation [8]. Viscosupplementation consists in the intra-articular injection of hyaluronic acid (HA), a naturally occurring joint component, which contributes to viscoelastic and mechanical properties of cartilage and synovial fluid (shock absorption, joint lubrication and cartilage protection [9–11]). As the molecular weight and concentration of endogenous HA are reduced during OA, viscosupplementation aims at restoring the metabolic and rheological homeostasis of the joint [12].

Since the initial hypothesis regarding the role of bone in OA pathogenesis formulated by Radin et al. [13], subchondral bone changes have been established as one of some possible factors in disease onset and progression. The nature of subchondral bone remodeling changes in OA [14] and the time frame in which they occur is not fully understood, particularly in the early-stage of the disease [15]. High molecular weight HA is widely used for its chondroprotective property [16], which preserves cartilage micro-structure but several studies reported contradictory effects of HA on bone. Pilloni et al. [17] showed in vitro human osteogenic potential, leading to osteoblast bone matrix protein expression. However, Prince et al. [18] observed in vivo osteoclast activation, leading to bone resorption. To date no clear action of HA has been depicted on bone and only few investigators have explored the effect of intra-articular HA injection on key determinants of subchondral bone microarchitecture and strength [19–21].

One currently used HA commercial product is Ostenil® which must be injected once a week for three to five weeks to get an effect lasting around six months. In order to improve the efficacy of HA within the articulation and reduce potential side-effects from the repetitive intra-articular injections, one approach consists in associating HA with a second biopolymer: chitosan. Chitosan (Cs) is a linear polysaccharide originated from a deacetylation of chitin, the primary structural polymer of the exoskeleton of marine invertebrates, insects, and cell wall of fungi [22]. Cs has been proven to be chondroprotective and to increase chondrocyte proliferation when injected intra-articularly in rat [23] and rabbit [24, 25] OA models. Cs was also shown to promote bone formation in porous Cs sponge in vitro [26] and osteoinduction in vivo [27, 28]. However, as Hoemann et al. [29, 30] pointed out, Cs increased initial resorption of microdrilled subchondral bone in rabbit knees. While Cs is a promising compound for viscosupplementation, as evidenced by its chondroprotection properties in preclinical studies, no study in ACLT rabbit model of OA has described Cs effects on subchondral bone yet.

The aim of the study was to investigate the effect of intra-articular injection of a novel hybrid hydrogel based on reacetylated fungal Cs added to HA on subchondral bone. Several microarchitectural parameters (subchondral plate thickness, trabecular thickness, trabecular separation, bone volume fraction) as well as bone mineral density and tissue mineral density were investigated. Final evaluations were performed six weeks after ACLT in rabbits (i.e. rabbits in the early-stage of the disease). More precisely, the hypothesis that the novel hybrid hydrogel compared to a high molecular weight HA commercial formulation would change subchondral bone microarchitectural parameters and diminish bone mineral density was tested. Such an early change on the subchondral bone might be symptomatic of an OA disease modification.

## Method

### Animal model

The experimental work on rabbits was performed under the authorization of the ethical committee of VetAgro Sup, veterinary campus of Lyon (authorization number 1373) and in full accordance with European legislation and the ARRIVE statement [31]. Eighteen healthy adult male white New Zealand rabbits (5 months of age, 3.68 ± 0.18 kg) were provided by Centre Lago (Vonnas, France). As described previously [32],after 2 weeks in acclimation and quarantine in individual boxes, experimental OA was surgically induced by unilateral ACLT performed by a trained veterinary surgeon. Before surgery, the animals received subcutaneous injections of 30 mg/kg Borgal® (sulfadoxine and trimethoprim) bid, 0.1 mg/kg morphine, and 0.4 mg/kg Meloxidyl® (meloxicam). Deep anaesthesia was induced by intra-muscular injection of 40 mg/kg Ketamine 1000® and 80 ml/kg Domitor® (medetomidine) and then maintained by 1–3.5% isoflurane administered via endotracheal intubation. After careful shaving and disinfection with Vetedine® (povidone iodine) soap and solution, ACLT was performed on the left leg with a lateral approach [7], while the right knee was not operated. The complete rupture of the ACL was assessed by the anterior drawer sign (manual horizontal dislocation) before the closure of the articular capsule. The operated leg was not immobilized, and rabbits were allowed to move freely in their individual cages after the surgery.

### Postoperative care

The rabbits received subcutaneous injection of 0.01 mg/kg buprenorphine bid for 4 days to avoid pain, 0.5 mg/kg Emeprid® (metoclopramide) for 3 days, 15 mg/kg Borgal® bid for 9 days and 1 cp/day Feligastryl® (eserine) for 3 days to reduce the risk of obstipation. Cothivet® spray was applied to the wound for 6 days after surgery. Veterinarians closely monitored the recovery, and a careful clinical follow-up was performed every other day. This postoperative care reduced pain and prevented lameness. All rabbits recovered fully from the surgery.

### Formulation administration

The operators of the injections and evaluations were blinded to the formulations. The 18 rabbits were randomized into 3 groups of 6 rabbits. Each ACLT knee (left knee) was treated by intra-articular injection of 0.2 ml of either Saline solution, HA commercial formulation (Ostenil®, TRB Chemedica, Switzerland), or novel hybrid hydrogel formulation (Cs added to HA) [25, 33, 34], respectively called hereafter Saline-group, HA-group and Hybrid-group. Eight non-operated right knees (randomly chosen within groups) were used as non-operated controls (referred hereafter to as Contralateral-group, *n* = 8). Intra-articular injections were performed at weeks 1, 2, 3, 4, and 5 post-ACLT after a short time of anaesthesia (40 mg/kg Ketamine 1000® and 80 μg/kg Domitor®) and careful disinfection (Vetedine® soap and solution).

### OA grading and micro-computed tomography imaging of subchondral bone

After a 6-week observation period, the rabbits were sacrificed by intra-vascular administration of 1 ml/kg Dolethal® (pentobarbital) after being chemically restrained by intra-muscular injection of 40 mg/kg Ketamine 1000® and 80 mg/kg Domitor®. Careful dissection of both knees and a saw section of the proximal part of the tibia was performed. Cartilage degradation, as well as osteophyte production, were graded using a macroscopic grading system developed by Laverty et al. [34]. To assess the stage of OA for each tissue, a mean value of OA score was calculated as an average of femoral and tibial grades prior to micro-computed tomography.

Full details on micro-computed tomography imaging technique on eXplore Locus system (General Electric, Fairfield, USA) are provided in our previous study [33]. The acquisition was performed with 45 μm^3^ isotropic resolution at 80 kV and 450 μA with a FOV 80 mm in diameter and 35 mm in depth. After acquisition and reconstruction, 16-bit images were calibrated with a phantom containing hydroxyapatite, water, and air and expressed in Hounsfield Units (HU: air 1000 HU and calcified tissues >100 HU).

### Microarchitectural parameter measurements

**Fig. 1:**
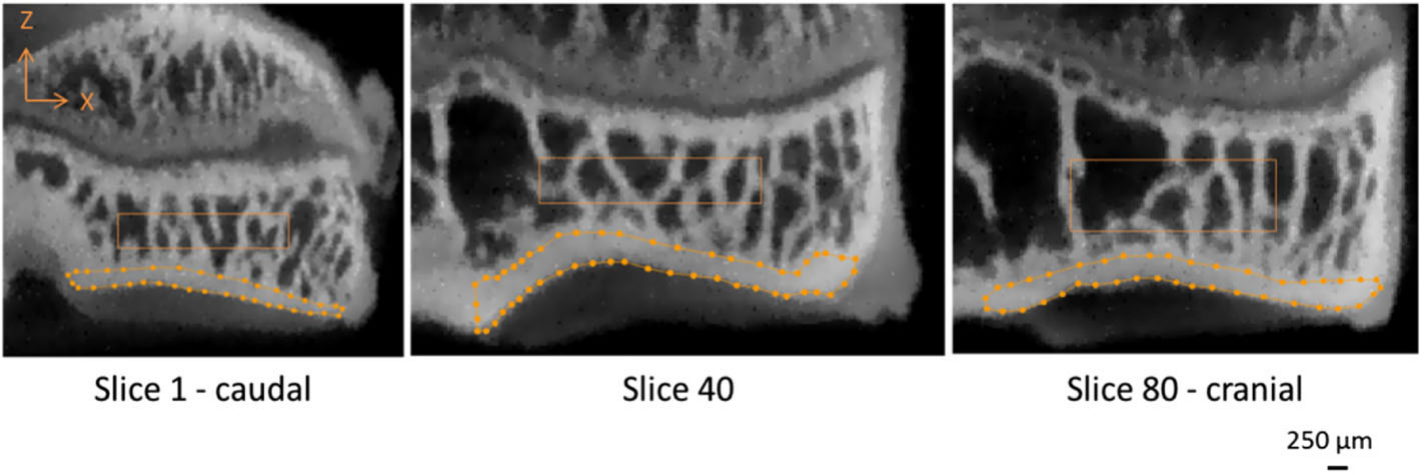
Medial tibial condyle ROIs at different locations in the Y (cranio-caudal) axis of subchondral bone plate and trabecular bone from Contralateral-group

Image treatment and parameters measurements were performed by a single operator blinded for the assessment using MicroView software ABA 2.2 (General Electric, Fairfield, USA). An anisotropic filter was applied to the images. Individual semi-automatic segmentation was performed based on Otsu method [35] to distinguish subchondral bone plate from trabecular bone. Subsequently, based on Hounsfield numbers, the region of interest (ROI) was defined manually using a contour-based tool on the weight-bearing area of the medial tibial condyle, as follows and illustrated in Fig. 1. Firstly, the X (medio-lateral) and Y (cranio-caudal) axes were set from the intercondylar area to the lateral edge of the cortical bone and from the intercondylar area to the caudal aspect to the medial condyle, respectively. Secondly, the Z axis (proximo-distal axis) was adapted to each tissue: subchondral bone plate, from the calcified cartilage to the end of the subchondral bone plate; and trabecular bone, from the subchondral bone plate/trabecular bone junction to the end of the epiphyseal line. Approximately 100 slices were delineated by interpolation for the subchondral cortical bone and approximately 80 for the trabecular bone. The difference in slice number was attributed to ROI of trabecular volume which became too small where the intercondylar area met the epiphyseal line.

The microarchitectural parameters were then calculated according to classical 2D histomorphometric method [36]: (i) subchondral bone plate: mean subchondral bone plate thickness (Pt.Th [mm]); (ii) trabecular bone: mean trabecular thickness (Tb.Th [mm]), mean trabecular separation (Tb.Sp [mm]) and mean trabecular bone volume fraction (Tb.BV/TV [%]). Moreover, mean bone mineral density (BMD [mg of mineral content per cc]) and mean bone tissue mineral density (TMD [mg of mineral content per cc]) were measured for both the subchondral bone plate, denoted Pt, and the trabecular bone, denoted Tb. For each voxel inside the ROI, 3D local thickness was obtained based on Hildebrand and Rüegsegger method [37].

### Microarchitectural and mineral density parameters statistical analysis

Statistical analysis was performed with R (R Foundation for Statistical Computing, Vienna, Austria version 3.1.2014–10-26) using the Kruskal-Wallis ANOVA test (α = 0.05) followed by the Mann-Whitney-Wilcoxon rank test (α = 0.05) as post hoc analysis when Kruskal-Wallis showed statistical significance. Firstly, Saline-group, HA-group and Hybrid-group were compared to Contralateral-group to assess the effects of ACLT on subchondral bone 6-weeks post-surgery, hereafter statistical significance is denoted by the symbol #. Secondly HA-group and Hybrid-group were compared to Saline-group to explore the viscosupplementation effects on subchondral bone, hereafter statistical significance is denoted by the symbol *. Finally, to test the combined effect of Cs and HA, Hybrid-group was compared to HA-group, in this case statistical significance is denoted by the symbol ∆.

## Results

**Fig. 2:**
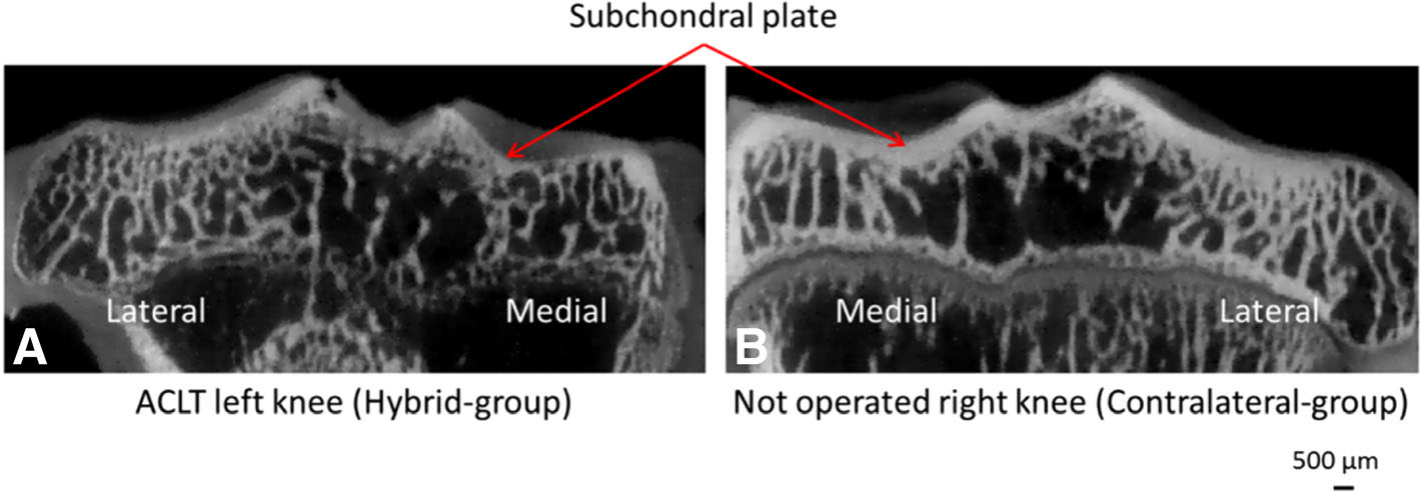
Typical ex vivo microtomography images of tibial plate (6 weeks after ACLT). **a** Hybrid-group (*left knee*); **b** Contralateral-group (*right knee*)

Mean OA scores obtained for Contralateral, Saline, HA and Hybrid groups were 0.45±0.11, 1.8±0.72, 2.14±0.62 and 1.53±0.3, respectively. By consequence, all operated groups exhibited early OA lesions, while contralateral knees showed no macroscopic sign of OA. Figure 2 shows typical micro-tomography images of tibial plates from Hybrid-group and Contralateral-group. Bone modifications were striking, in particular the subchondral bone plate thickness was importantly reduced in Hybrid-group (Fig. 2). To confirm and measure this observation, subchondral bone microarchitectural parameters and mineral density were investigated quantitatively for all groups (Table 1).

**Table 1:** Microarchitectural parameters of medial and lateral tibial condyles: cartilage (C), subchondral bone plate (Pt) and trabecular bone (Tb) at 6 weeks post ACLT and their statistical significance compared to Contralateral-group (#), Saline-group (*) or HA-group (∆)

Contralateral-group (*n* = 8)			Saline-group (*n* = 6)				HA-group (*n* = 6)						Hybrid-group (*n* = 6)							
Parameters	Medial	Lateral	Medial	*P-values*	Lateral	*P-values*	Medial	*P-values*		Lateral	*P-values*		Medial	*P-values*			Lateral	*P-values*		
	Mean ± SD	Mean ± SD	Mean ± SD	*#*	Mean ± SD	*#*	Mean ± SD	*#*	*	Mean ± SD	#	*	Mean ± SD	*#*	*	∆	Mean ± SD	*#*	*	∆
C.Th [mm]	0,66 ± 0,03		0,57 ± 0,04				0,64 ± 0,10						0,61 ± 0,05							
Pt.TH [mm]	0,40 ± 0,04	0,44 ± 0,07	0,36 ± 0,04		0,35 ± 0,11		0,39 ± 0,06			0,38 ± 0,08			0,27 ± 0,06	0,008		0,009	0,22 ± 0,03	0,008		0,009
Pt.BMD [mg/cm^3^]	621,6 ± 21,0	608,8 ± 33,3	610,7 ± 28,0		602,1 ± 46,8		558,3 ± 28,2	0,001	0,026	559,2 ± 37,7	0,001	0,026	524,7 ± 39,7	0,001	0,009		554,4 ± 24,7	0,001	0,009	
Pt.TMD [mg/cm^3^]	622,8 ± 21,1	629,0 ± 34,6	616,1 ± 29,2		652,3 ± 28,5		561,6 ± 25,8	0,001	0,026	599,2 ± 28,0	0,001	0,026	545,1 ± 37,5	0,001	0,026		601,6 ± 22,2	0,001	0,026	
Tb.Th [mm]	0,16 ± 0,02	0,17 ± 0,02	0,14 ± 0,01		0,18 ± 0,02		0,14 ± 0,02			0,19 ± 0,02			0,12 ± 0,01	0,001	0,009	0,026	0,16 ± 0,01	0,001	0,009	0,026
Tb.Sp [mm]	0,27 ± 0,04	0,24 ± 0,04	0,29 ± 0,05		0,26 ± 0,04		0,33 ± 0,05	0,043		0,28 ± 0,05	0,043		0,32 ± 0,02	0,043			0,28 ± 0,02	0,043		
Tb.BV/TV [%]	0,49 ± 0,05	0,53 ± 0,03	0,44 ± 0,03		0,53 ± 0,02		0,40 ± 0,02	0,005		0,51 ± 0,03	0,005		0,36 ± 0,05	0,003	0,026		0,46 ± 0,03	0,003	0,026	
Tb.BMD [mg/cm^3^]	387,8 ± 43,6	378,6 ± 25,1	319,5 ± 26,0	0,029	370,0 ± 23,9	0,029	284,3 ± 29,0	0,003		337,0 ± 15,9	0,003		237,5 ± 28,5	0,001	0,004	0,026	309,2 ± 15,8	0,001	0,004	0,026
Tb.TMD [mg/cm^3^]	553,2 ± 30,8	516,0 ± 22,3	497,2 ± 24,5	0,008	511,1 ± 18,4	0,008	473,7 ± 30,2	0,003		484,4 ± 21,6	0,003		418,2 ± 15,4	0,001	0,002	0,015	462,2 ± 17,3	0,001	0,002	0,015

#*P*-values showing significant difference with Contralateral-group (Wilcoxon rank, *p* < 0.05)

*P-values showing significant difference with Saline-group (Wilcoxon rank, *p* < 0.05)

∆*P*-values showing significant difference with HA-group (Wilcoxon rank, *p* < 0.05)

Firstly, cartilage thickness (C.Th), subchondral bone plate thickness (Pt.Th), trabecular bone thickness (Tb.Th), trabecular separation (Tb.Sp) and trabecular bone volume fraction (Tb.BV/TV) mean values were measured.

Secondly, bone mineral density (BMD) and tissue mineral density (TMD) mean values were measured in both subchondral bone plate (Pt) and trabecular bone (Tb).

### Comparison to contralateral-group

**Fig. 3:**
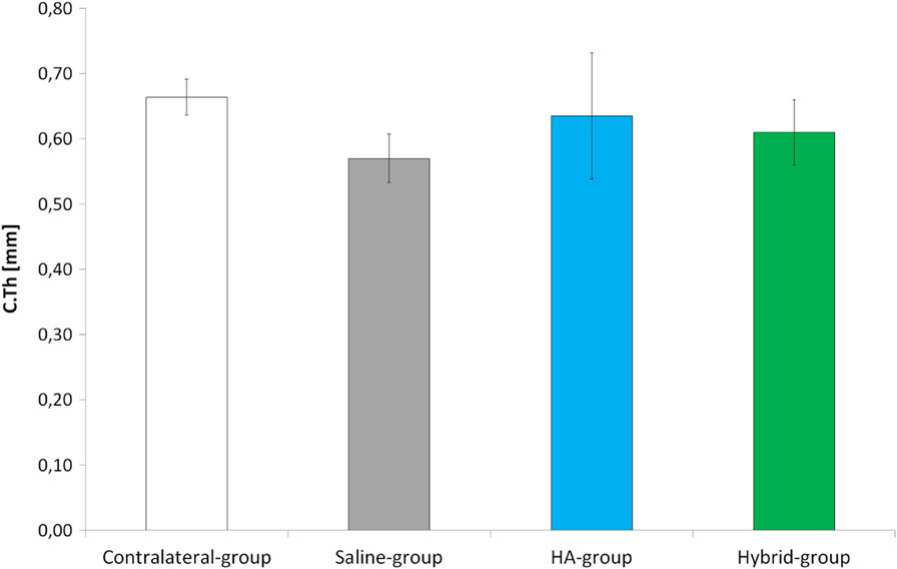
Cartilage thickness (mean ± SD) for Contralateral, Saline, HA and Hybrid groups. Statistical significance compared to Contralateral-group is indicated by # (i.e.*,* pairwise Wilcoxon Rank test, α = 0.05)

C.Th mean value (Fig. 3) was significantly lower in Saline-group than in Contralateral-group (*p* = 0.002). No significant differences in C.Th mean values were observed in HA-group and Hybrid-group compared to Contralateral-group.

**Fig. 4:**
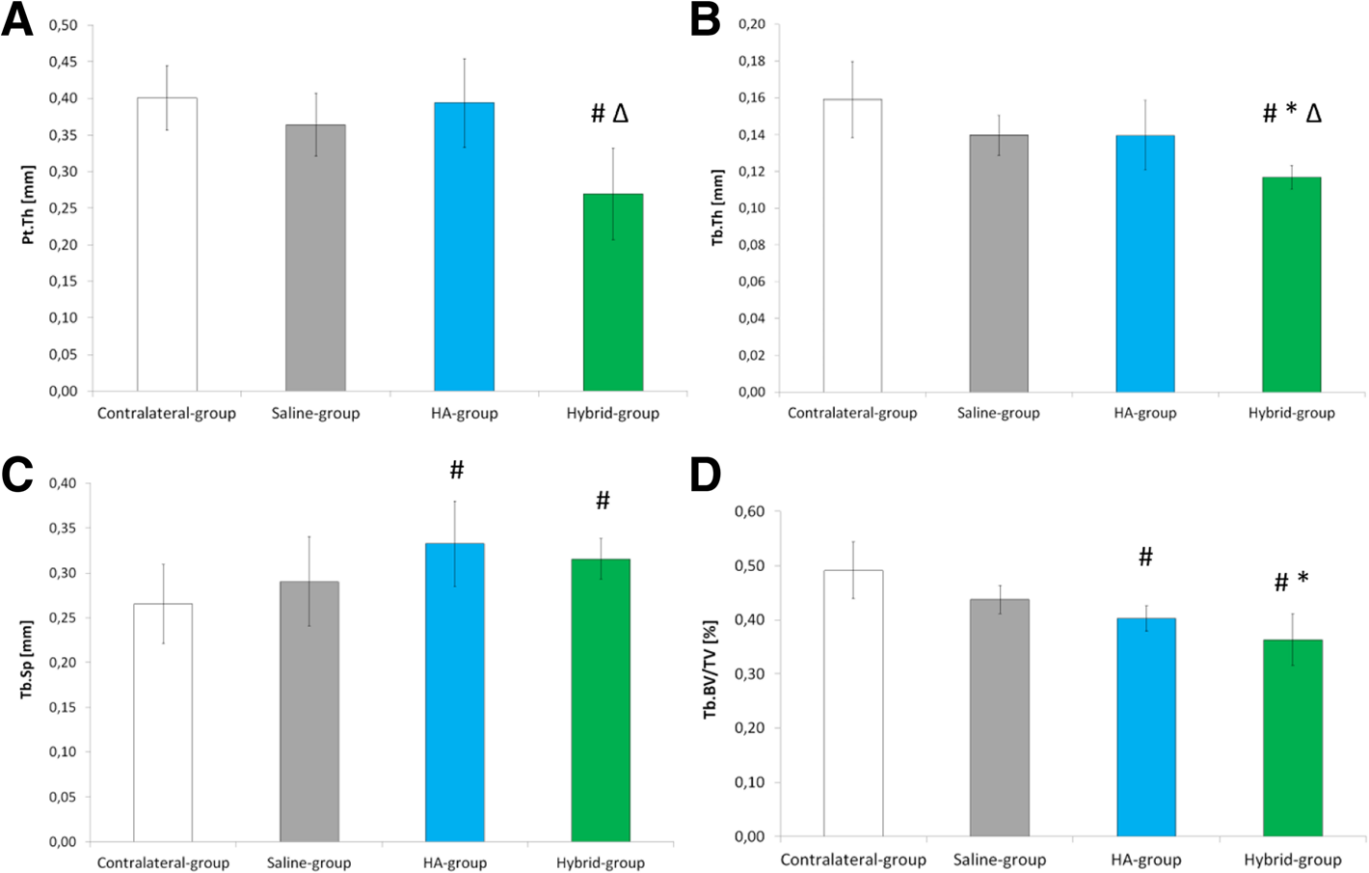
Microarchitectural parameters (mean ± SD) in Contralateral, Saline, HA and Hybrid groups. **a** subchondral bone plate thickness (Pt.Th); **b** trabecular thickness (Tb.Th); **c** trabecular separation (Tb.Sp); **d** trabecular bone volume fraction (Tb.BV/TV). #, * and ∆ indicate statistical significance under pairwise Mann-Whitney-Wilcoxon Rank test (α = 0.05) compared to Contralateral-group, Saline-group and HA-group, respectively, performed after significant Kruskall-Wallis ANOVA test (α = 0.05)

Bone microarchitectural parameters are displayed in Fig. 4.

In Saline-group compared to Contralateral-group, no significant differences were observed in Pt.Th, Tb.Th, Tb.Sp and Tb.BV/TV mean values. In HA-group compared to Contralateral-group, Tb.Sp mean value was significantly increased and Tb.BV/TV mean value was significantly decreased (*p* = 0.043 and *p* = 0.005, respectively). In Hybrid-group compared to Contralateral-group, bone parameter mean values were all significantly modified, with an increase in Tb.Sp (*p* = 0.043) and a decrease in Pt.Th, Tb.Th and Tb.BV/TV (*p* = 0.008, *p* = 0.001 and *p* = 0.003, respectively).

**Fig. 5:**
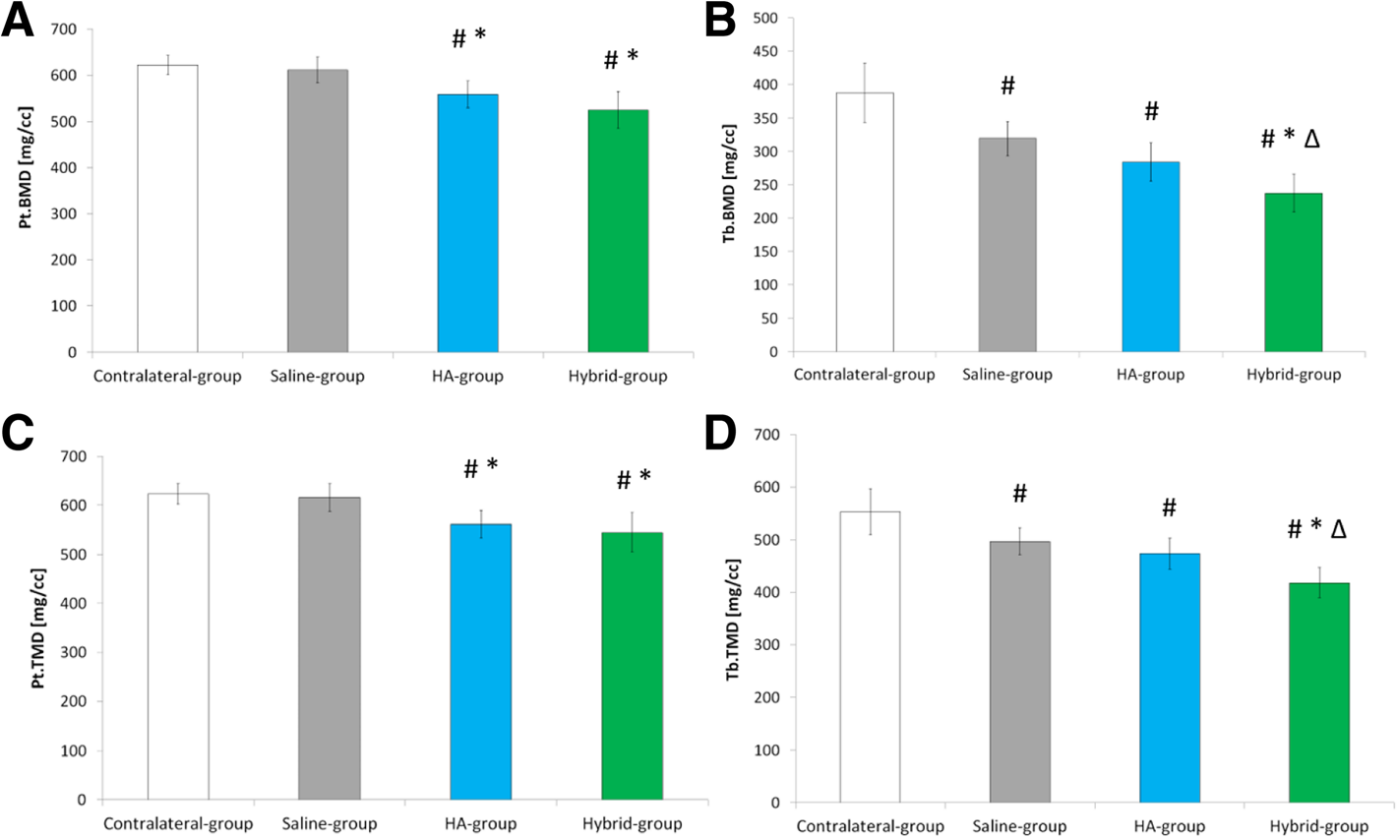
Bone Mineral Density (mean ± SD) in Contralateral, Saline, HA and Hybrid groups. **a** subchondral bone plate BMD (Pt.BMD); **b** trabecular bone BMD (Tb.BMD), **c** subchondral bone plate TMD (Pt.TMD); **d** trabecular bone TMD (Tb.TMD). #, * and ∆ indicate statistical significance under pairwise Mann-Whitney-Wilcoxon Rank test (α = 0.05) compared to Contralateral-group, Saline-group and HA-group, respectively, performed after significant Kruskall-Wallis ANOVA test (α = 0.05)

Mineral densities (Pt.BMD, Tb.BMD, Pt.TMD, Tb.TMD) mean values are displayed in Fig. 5.

Regarding bone mineral density, in Saline-group compared to Contralateral-group, Tb.BMD and Tb.TMD mean values were significantly decreased (*p* = 0.29 and *p* = 0.008, respectively) (Fig. 5B-D). In both HA-group and Hybrid-group compared to Contralateral-group, Pt.BMD mean values were significantly decreased (*p* = 0.001 and *p* = 0.001, respectively) (Fig. 5A) and Tb.BMD mean values were also significantly decreased (*p* = 0.003 and *p* = 0.001, respectively) (Fig. 5B).

Regarding tissue mineral density, in Saline-group compared to Contralateral-group Pt.TMD and Tb.TMD mean values were significantly decreased (*p* = 0.029 and *p* = 0.008) (Fig. 5B-D). In both HA-group and Hybrid-group compared to Contralateral-group, Pt.TMD mean values were significantly decreased (*p* = 0.001 and *p* = 0.001, respectively) (Fig. 5C) and Tb.TMD mean values were also significantly decreased (*p* = 0.003 and *p* = 0.001, respectively) (Fig. 5C).

### Comparison to saline-group

No significant differences in C.Th mean value (Fig. 3) were observed in HA-group and Hybrid-group compared to Saline-group.

Concerning subchondral bone microarchitectural parameters, in HA-group compared to Saline-group, no significant differences were observed in microarchitectural bone parameters (Pt.Th, Tb.Th, Tp.Sp and Tb.BV/TV). In Hybrid-group compared to Saline-group, Tb.Th and Tb.BV/TV mean values were significantly decreased (*p* = 0.009 and *p* = 0.026, respectively) (Fig. 4).

Regarding bone mineral density (Fig. 5), in HA-group compared to Saline-group, Pt.BMD and Pt.TMD mean values were significantly different (*p* = 0.026 and *p* = 0.026 respectively). In Hybrid-group compared to Saline-group, Pt.BMD, Pt.TMD, Tb.BMD, and Tb.TMD mean values were all significantly decreased (*p* = 0.009, *p* = 0.026, *p* = 0.004 and *p* = 0.002, respectively).

### Comparison between HA-group and hybrid-group

C.Th mean value showed no differences between HA and Hybrid groups (Fig. 3).

Concerning subchondral bone microarchitectural parameters in Hybrid-group compared to HA-group (Fig. 4) Pt.Th and Tb.Th mean values were significantly decreased (*p* = 0.009 and *p* = 0.026, respectively).

Regarding bone mineral density (Fig. 5), Tb.BMD and Tb.TMD mean values were significantly decreased in Hybrid-group compared to HA-group (*p* = 0.026, *p* = 0.015 respectively).

Finally, bone microarchitectural parameters and bone mineral density mean values on lateral tibial condyle exhibited same significant changes as medial tibial condyle (Table 1).

## Discussion

In this study, the effect of the addition of Cs to HA on OA subchondral bone during viscosupplementation was investigated. The novel hybrid hydrogel constituted of reacetylated fungal Cs added to HA was compared to a high molecular weight HA commercial formulation. Subchondral bone microarchitectural parameters and mineral density were measured 6 weeks post-ACLT in rabbits. Our results support the hypothesis that Cs contributes to subchondral bone changes and leads to increased subchondral bone loss in early OA in rabbits. The effect on cartilage and especially cartilage histology has also been evaluated and published by Kaderli et al., [33].

A significant decrease of C.Th mean value confirming OA induction was observed when the Saline-group was compared to the Contralateral-group, while no significant changes were observed for all the bone parameters. Contrastingly, no significant decrease of C.Th mean value was observed when the HA and Hybrid-groups were compared to the Contralateral-group suggesting a preserved chondroprotective effect of HA when combined to Cs. Those results are in accordance with other studies [19, 38, 39].

Notably, the present study highlights subchondral bone loss following HA-based viscosupplement therapy in OA joints. On the one hand, literature show that during OA, intra-articularly injected high-molecular weight HA penetrates into the OA affected subchondral bone reaching the bone marrow space and osteocyte lacunae, where it can enhance intrinsic HA synthesis [38]. On the other hand, intrinsic HA content of bone extracellular matrix is closely linked to bone homeostasis in general and to osteoclast-mediated bone resorption in particular [18]. In the present study, the decrease in subchondral bone microarchitectural parameters was associated with demineralization, which suggests subchondral bone resorption, likely as a result of the effect of HA on osteoclast activity in OA subchondral bone [38]. On the contrary, in a rabbit model of OA, Permuy et al. [21] did not observe any significant subchondral bone loss following HA intra-articular injection in a OA rabbit model [21]. This discrepancy might result from the more severe induction technic (combining ACLT and meniscectomy vs. ACLT) and the later endpoint used in their study (11 vs. 6 weeks) leading to a more advanced OA and thus to the recovery of initial subchondral bone microarchitectural parameters and mineral density [5, 15].

The intra-articular injection of Cs added to HA (Hybrid-group) significantly decreased several subchondral bone microarchitectural parameters as compared to intra-articular injection of saline and HA. In particular, trabecular thickness, trabecular bone volume fraction, and bone mineral densities mean values (Fig. 4 and Fig. 5) were reduced as compared to the Saline-group, as well as subchondral bone plate thickness, trabecular thickness and trabecular bone mineral density mean values compared to HA-group. Previous studies showed that, similarly to HA, Cs mediates osteoclast cells adhesion to subchondral bone promoting resorption [39]. Our results confirm the role of Cs on subchondral bone loss, as observed by Hoemann et al. [29, 30] on microdrilled rabbit knee defect filled with Cs-based implant. Interestingly, no loss in mineralization was observed in the subchondral bone plate (i.e. cortical bone) in contrast with the subchondral trabecular bone (Fig. 5), which can be explained by the lower cortical bone remodeling rate [40] (up to tenfold) compared to trabecular bone.

In the present study, OA induction was verified by X-ray osteophyte scoring on all operated knees. The absence of such was also verified on all contralateral knees [33]. In order to avoid group’s size discrepancy, a “Contralateral-group” has been formed with randomly chosen contralateral legs from each treated group. This approach has been used in numerous studies [6, 41, 42, 43]. Moreover, the same significant changes were found whether the medial or lateral condyles were investigated. As well, when lateral condyles were compared to medial condyles within each group, there were no significant differences within Contralateral-group, Saline-group, HA-group and Hybrid-group. Thus, equilibrated contact-loads on tibial condyles are suggested.

Viscosupplementation with high molecular weight HA significantly decreased subchondral bone mineral density as compared to saline solution. Our study also shows that Cs added to HA enhanced subchondral bone loss as compared to HA alone by decreasing significantly subchondral bone parameters and bone mineral density. In order to go one step further in the evaluation of the effect of Cs on subchondral bone, the impact of bone microarchitectural parameters on the mechanical compliance as well as their relationship with the cartilage integrity is going to be evaluated.

## Conclusion

This study demonstrates that the commercial formulation of HA injected intra-articularly induces subchondral bone demineralization in the early-stage of OA induced by ACLT in rabbits. Moreover, Cs added to HA enhances this subchondral bone loss compared to HA alone.. To the authors’ knowledge, this is the first study investigating the effects of Cs added to HA on subchondral bone microarchitectural parameters and mineral density in experimental OA.
